# Supplementary material for: Gender Difference in Fear and Anxiety about and Perceived Susceptibility to COVID-19 in the Third Wave of Pandemic among the Japanese General Population: A Nationwide Web-Based Cross-Sectional Survey
Source: Int J Environ Res Public Health. 2022 Dec 4;19(23):16239. doi: 10.3390/ijerph192316239 (PMC9738986; doi:10.3390/ijerph192316239)
Supplement: Supplementary file 1 [file ijerph-19-16239-s001.zip › ijerph-2006998-supplementary.pdf]

Supplementary Table S1. Standards of effect sizes

Test	Indicator	Effect size		
		Small	Medium	Large
t-test	d	0.2	0.5	0.8
Analysis of variance	η^2	0.01	0.06	0.14
Chi-square test (2 x 2)	ϕ	0.1	0.3	0.5
Chi-square test (larger than 2 x 2)	V	0.1	0.3	0.5

Supplementary Table S2. Mean (standard deviation: SD) of Fear of COVID-19 Scale score: sensitivity analysis (n = 28,000)

	Mean (SD)	P-value	Effect size
Sex			
Men	17.5 (5.9)	<0.001	0.188
Women	18.6 (5.6)		

Effect size is expressed by Cohen's d.

Supplementary Table S3. Standardized beta for Fear of COVID-19 Scale scores: multiple linear regression analysis, sensitivity analysis (n = 28,000).

	Standardized beta
Sex	
Men	Reference
Women	0.091***

All independent variables were included in the multiple linear regression model calculating adjusted odds ratios. ***: p<0.001.

Supplementary Table S4. Proportions of reporting a median or higher Fear of COVID-19 Scale score: sensitivity analysis (n = 28,000).

	N (%)	P-value	Effect size
Sex			
Men	6817 (51.4%)	<0.001	0.060
Women	7319 (57.4%)		

Effect size is expressed by ϕ.

Supplementary Table S5. Odds ratios for reporting a median or higher Fear of COVID-19 Scale score: multiple logistic regression analysis, sensitivity analysis (n = 28,000)

	**Odds Ratio (95% Confidence Interval)**	
	**Crude**	**Adjusted[1]**
Sex		
Men	Reference	Reference
Women	1.27 (1.21–1.34) ***	1.24 (1.18 – 1.31)***

[1]All independent variables were included in the multiple logistic regression model for calculating adjusted odds ratios. ***: p<0.001.

Supplementary Table S6. Proportions of perceived susceptibility to COVID-19: sensitivity analysis (n = 28,000)

	N (%)	**P-value**	**Effect size**
Sex			
Men	1562 (11.8%)	<0.001	0.033
Women	1782 (14.0%)		

Effect size is expressed by ϕ.

Supplementary Table S7. Odds ratios for perceived susceptibility to COVID-19: multiple logistic regression analysis, sensitivity analysis (n = 28,000)

	**Odds Ratio (95% Confidence Interval)**	
	**Crude**	**Adjusted[1]**
Sex		
Men	Reference	Reference
Women	1.22 (1.13–1.31) ***	1.26 (1.17 – 1.37)***

[1]All independent variables were included in the multiple logistic regression model for calculating adjusted odds ratios. ***: p<0.001.

Supplementary Table S8. Age and having a spouse (n = 23,516).

	Having a spouse	
	N (%)	**P-value**
Age		
15 – 19	18 (2.9%)	<0.001
20 – 29	520 (18.4%)	
30 – 39	1938 (59.7%)	
40 – 49	2804 (61.1%)	
50 – 59	2853 (66.7%)	
60 – 69	3334 (77.3%)	
70 - 80	2802 (76.7%)	

P-value was calculated with chi-square test.
